# The antibiotic de-escalation strategy in patients with multidrug-resistant bacterial colonization after allogeneic stem cell transplantation

**DOI:** 10.3389/fmicb.2024.1487617

**Published:** 2025-01-03

**Authors:** Roberto Bono, Giuseppe Sapienza, Stefania Tringali, Cristina Rotolo, Alessandra Santoro, Laura Di Noto, Angelo Pirrera, Floriana Schirò, Raffaella Rubino, Antonio Cascio, Sergio Siragusa, Carmen Tommaselli, Orazia DiQuattro, Caterina Patti, Luca Castagna

**Affiliations:** ^1^BMT Unit, Azienda Ospedaliera Villa Sofia-Cervello, Palermo, Italy; ^2^Onco-hematology and Cell Manipulation Laboratory Unit, AOR Villa Sof ia-Vincenzo Cervello, Palermo, Italy; ^3^Transfusional and Transplantation Unit, Azienda Ospedaliera Villa Sofia-Cervello, Palermo, Italy; ^4^Department of Health Promotion, Mother and Child Care, Internal Medicine and Medical Specialties "G. D'Alessandro", University of Palermo, Palermo, Italy; ^5^Department PROMISE-Infectious and Tropical Diseases Unit, AOU Policlinico "P. Giaccone", University of Palermo, Palermo, Italy; ^6^Hematology Department Ospedale Civico, Palermo, Italy; ^7^Microbiological Unit, Azienda Ospedaliera Villa Sofia-Cervello, Palermo, Italy; ^8^Department of Hematology, Azienda Ospedaliera Villa Sofia-Cervello, Palermo, Italy

**Keywords:** MDR, allogeneic stem cell transplantation, de-escalation antibiotic therapy, stool colonization, empiric antibiotic therapy

## Abstract

Colonization by multidrug-resistant (MDR) bacteria and related bloodstream infections (BSI) are associated with a high rate of mortality in patients with hematological malignancies after intensive chemotherapy and allogeneic stem cell transplantation (allo-SCT). In this retrospective study, we analyzed the outcomes of patients colonized with MDR bacteria (primarily carbapenem-resistant *klebsiella pneumoniae*, KPC), before allo-SCT. We also investigated the feasibility and safety of an antimicrobial de-escalating approach in these patients. Since 2021, 106 patients have been undergoing allo-SCT in our department, and 34 (32%) of them were colonized by MDR bacteria before allo-SCT. In the pre-engraftment period, 84% received an empiric antibiotic therapy (EAT) active against MDR bacteria and 16% were treated with a conventional EAT. The MDR translocation rate was null, and the overall de-escalation rate was 79%, with 75% in patients with fever of unknown origin (FUO). Among the cohort of patients with MDR-positive rectal swabs just before allo-SCT (*n* = 18), the de-escalation rate was 100%. The all-cause mortality rates at 30 and 100 days for the whole MDR patient population were 6% (2/34) and 12% (4/34), respectively. Day +30 infection-related mortality rate was 3%. In this study, we confirm the safety of the de-escalation approach in patients with previous MDR infection after allo-SCT. This could reduce the exposure time to EAT antibiotics, reducing the selective pressure.

## Introduction

Allogeneic stem cell transplantation (allo-SCT) is still considered the only curative therapy for several hematological diseases. However, allo-SCT carries *per se* a high risk of morbidity and mortality, due to toxicities mainly linked to graft versus host disease (GVHD) and infections. Furthermore, in the past decade, the number of elderly patients over 65 years of age undergoing allo-SCT has steadily increased. Complications are high in this patient population, and age is a negative prognostic factor. While the introduction of more effective GVHD prophylaxis, such as post-transplantation cyclophosphamide (PTCY) or the increased use of anti-thymocyte globulin (ATG) in the context of HLA identical donors, has reduced the incidence of acute and chronic GVHD, infectious complications, especially those caused by multidrug-resistant (MDR) bacteria, have become an emerging issue (Averbuch et al., 2017). From this point of view, allografted patients are considered a very high-risk population because of the contemporary presence of multiple risk factors such as neutropenia and gastrointestinal mucositis during the pre-engraftment period, and persisting humoral and cellular immunodeficiency due to immunosuppressive drugs, GVHD, and its therapy.

The standard of care for neutropenic patients with fever is to start empirical antibiotic therapy (EAT), mainly against gram-negative bacteria that may translocate from the gastrointestinal tract. While this strategy is well-established and considered the standard of care in patients with NF (Pizzo, 1993), the optimal duration of antibiotic therapy during the aplasia period remains unclear. In the adult population, several studies have demonstrated that discontinuing or de-escalating EAT to fluoroquinolone prophylaxis in patients who have been afebrile for at least 48–72 h, without clinical/microbiological documentation, while still experiencing persistent severe neutropenia, is both feasible and safe (Winget et al., 2024; Petteys et al., 2020; Aguilar-Guisado et al., 2017). However, in these studies, the number of patients receiving allo-SCT was limited and the presence of those colonized with MDR was not reported. Thus, the efficacy and safety of EAT de-escalation in patients colonized by MDR with fever during the pre-engraftment period was not firmly established.

Colonization by MDR bacteria and related bloodstream infections (BSI) are associated with a high rate of mortality, reported to be up to 64%, in patients with hematological malignancies after intensive chemotherapy and allo-SCT (Girmenia et al., 2015; Patriarca et al., 2017; Forcina et al., 2018). Carbapenemase-producing enterobacteriaceae (CPE) represents a major challenge for clinicians, a serious problem for public health, and shows a high rate of resistance further to common antibiotics (ATB). Moreover, aplasia, the use of broad-spectrum ATB, and gut barrier damage result in a high risk of microbial translocation into the bloodstream, exposing patients to life-threatening systemic infections.

Several guidelines deal with the approach to managing patients with MDR bacterial infections. The European Conference on Infection in Leukemia (ECIL) guidelines suggested that in neutropenic patients with fever, an escalation or de-escalation approach can be adopted based on several considerations. In particular, for patients colonized with MDR bacteria, it is suggested that EAT adjusted to MDR should be promptly started, adopting a de-escalation strategy in the absence of microbiological documentation, and initial ATB therapy should be revised (Averbuch et al., 2013a). Recently, the European Society of Clinical Microbiology and Infectious Diseases (ESCMID) published guidelines for the treatment of MDR in the era of novel antibiotics active against difficult-to-treat microorganisms. However, even if the ESCMID guidelines did not consider allografted patients colonized with MDR bacteria, judicious use of broad-spectrum antibiotics is strongly claimed to try to avoid the development of resistance (Paul et al., 2022).

Recently, new combinations with penicillin plus Beta-lactamase inhibitors or newer ATB are increasingly used for the treatment of MDR infections during febrile neutropenia episodes. The reason behind this lies in the evidence of colonized patients with a high mortality rate who were not treated with empirically adapted antibiotics (Micozzi et al., 2021). Although such ATB are active against several MDR strains, a major concern is the development of resistant bacteria. In this scenario, it has been suggested that a de-escalation approach could reduce the selective pressure limiting the risk of ultra-multiresistant strain appearance (Pasquau-Liaño et al., 2022). In a recent prospective study from GITMO, the incidence of gram-negative infections was 17% in the first 30 days after allogeneic stem cell transplantation (allo-SCT). Colonization by carbapenem-resistant *klebsiella pneumoniae* (KPC) was a strong risk factor for pre-engraftment KPC infection due to gut translocation during the aplasia period. In this patient population, the frequency of pre-allo-SCT KPC colonization was 3.4% (Girmenia et al., 2017).

In a retrospective study, including 15% of patients with leukemia or cancer, an appropriate therapy against CPE (starting less than 5 days) was a protective factor, reducing the risk of death. Similarly, combination therapy was protective only in high-risk patients (such as leukemic or cancer patients) (Gutiérrez-Gutiérrez et al., 2017). The efficacy of empirical MDR-directed antibiotic therapy has been recently suggested in a retrospective study, including 55 MDR-colonized patients with hematological malignancies, mostly treated with conventional chemotherapy, and only 12 allografted patients (Micozzi et al., 2023).

This retrospective study aims to analyze the outcomes of MDR-colonized patients before allo-SCT, with a special focus on those treated with active MDR-directed antibiotics during the first febrile episode and a de-escalation approach, provided blood cultures were negative.

## Patients and methods

In this retrospective study, we analyzed the outcomes of patients with MDR-bacteria colonization who underwent allo-SCT. For such patients, starting from 2021, we adopted the de-escalation approach. The strategy consisted of starting an empiric antibiotic therapy (EAT) with ATB active against MDR strains (ceftazidime–avibactam or meropenem–vaborbactam, according to ATB susceptibility) during the first neutropenic febrile episode (NFE), and de-escalation to conventional antibiotics (piperacillin–tazobactam, cefepime) after 48 h, provided blood cultures were negative for MDR bacteria.

No patients received fluoroquinolone prophylaxis.

### MDR-colonized definition

We defined MDR-colonized patients with the following characteristics:

- detection of MDR colonization in stool at any time during the conventional treatment.
- systemic infections sustained by MDR bacteria at any time during the induction or consolidation chemotherapy.
- detection of MDR bacteria in swabs during the work-up immediately before allo-SCT.

Inclusion criteria were: age > 18 years, colonization (in any site, at any time) by MDR bacteria before allo-SCT, or allo-SCT from any donor, any conditioning regimen, any stem cell source, and treatment with a combination of antibiotics active against MDR bacteria during the first febrile episode. A de-escalation approach was initiated, provided blood cultures were negative after 48 h and patients were clinically stable.

### De-escalation approach definition

The de-escalation strategy consisted of initial empirical treatment with MDR-directed antibiotics, followed by a switch to a broad-spectrum antibiotic, such as piperacillin–tazobactam or cefepime.

### Transplant procedures

Patients received transplants from any donor and the conditioning regimens were established according to the type of disease, disease status, and patient characteristics. The graft versus host disease (GVHD) prophylaxis was post-transplant cyclophosphamide-based or ATG-based, depending on the donor type. All patients received peripheral blood stem cells.

The pre-engraftment period started from the infusion of stem cells to the first 3 days with the absolute neutrophil count (ANC) equal to greater than 0.5 × 10^9^/L.

### GVHD diagnosis and treatment

Acute GVHD (aGVHD) was graded according to the Keystone criteria (Przepiorka et al., 1995), and chronic GVHD (cGVHD) was retrospectively graded by following the NIH criteria (Filipovich et al., 2005). Severe aGVHD forms (defined as grade ≥ 2) were treated with steroids as per local procedures. Moderate-to-severe cGVHD was treated with steroids by following an alternating schedule.

### Objectives and end-points

The main aim of this study was to describe the outcomes of MDR-colonized patients during the pre-engraftment period after allo-SCT and to investigate the efficacy and safety of the de-escalation approach in a cohort of patients with MDR-bacteria colonization, receiving an EAT that is active against resistant strains during the first neutropenic febrile episode (NFE). The primary end-point is the MDR sepsis-attributable mortality rate (AMR) in de-escalated patients. The secondary objectives of this study are the feasibility of de-escalation, the MDR-directed ATB duration, the survival, and the GVHD incidence.

### Statistical analysis

With regard to the categorical variables, the differences between the groups were assessed using the chi-squared test. Overall, the survival and progression-free survival analysis were performed through Kaplan–Meier estimates and the log-rank test. Cumulative incidence (CI) of aGVHD and NRM was based on the estimates of cumulative incidence and gray test. All data were recorded on an Excel data sheet and all the analyses, including the descriptive ones, were carried out using NCSS 2019 software.

## Results

This retrospective study included 106 patients undergoing allo-SCT between June 2021 and December 2023. Of these patients, 34 (32%) had MDR-bacteria colonization before allo-SCT. The patient characteristics for the whole cohort and for the colonized patients are reported in Table 1.

**Table 1:** Patient characteristics.

N	106 (100%)	34 (32%)	18 (17%)	*p*
**Cohort**	All patients	MDR cohort	MDR-colonized at allo-SCT	
M/F	61/45	23/11	13/5	0.04
**Median age (y)**	52 (range)	58 (range 19–74)	59 (range 20–74)	0.5
**Disease**				
Acute myeloid leukemia	56 (53%)	25 (74%)	16 (89%)	
Acute lymphoblastic leukemia	20 (19%)	5 (15%)	1 (5%)	
Myelodisplastic syndrome	8 (8%)	/	1 (5%)	
Lymphoma	9 (8%)	1 (3%)	/	
Myeloproliferative neoplasms	10 (9%)	1 (3%)	/	
Multiple myeloma	1 (1%)	/	/	
Blastic plasmocytoid dendritic cell neoplasm	1 (1%)	1 (3%)	/	0.1
Aplastic anemia	1 (1%)	1 (3%)	/	
**Disease status**				1
CR	79 (75%)	27 (79%)	15 (83%)	
No CR	27 (25%)	7 (21%)	3 (17%)	
HCT-CI*	42 (41%)	8 (24%)	4 (22%)	0.36
0–1	22 (22%)	10 (30%)	5 (28%)	
2	38 (37%)	16 (46%)	9 (50%)	
≥3				
**Donor**				0.94
HAPLO	38 (36%)	16 (47%)	6 (33%)	
MUD	33 (31%)	8 (24%)	5 (28%)	
HLAid	19 (18%)	7 (21%)	4 (22%)	
mMUD	16 (15%)	3 (9%)	3 (17%)	
**Stem cell source**				1
PBSC	103 (97%)	33 (99%)	18 (100%)	
BM	3 (3%)	1 (1%)	/	
**Conditioning regimens**				0.13
MAC	79 (73%)	23 (68%)	13 (72%)	
RIC	29 (27%)	11 (32%)	5 (28%)	
**GVHD prophylaxis**				0.76
CSA + MTX + ATG	44 (42%)	23 (68%)	12 (58%)	
PTCY+CSA + MMF	62 (58%)	11 (32%)	6 (42%)	

*HCT-CI was available for 33 patients in the MDR cohort and 102 in the whole cohort. CR, complete remission; Haplo, haploidentical; MUD, matched unrelated donor; mMUD, mis-matched unrelated donor; PBSC, peripheral blood stem cell; BM, bone marrow; MAC, myeloablative conditioning regimen; CSA, cyclosporine A; MTX, methotrexate; ATG, anti-thymocyte globulin; PTCY, post-transplant cyclophosphamide; MMF, mycofenolate mofetil.

### Main outcomes for the whole population

The median time to engraft (defined as ANC 0.5 × 10^9^/L) was 17 days (range 12–32). The median time to safe platelet count (platelet count more than 20 × 10^9^/L) was 20 days (range 9–94). The 3-CI of grade 2–4 and 3–4 aGVHD was 20 and 7%, respectively.

The 100-day and the 1-year NRM CI were 13 and 22%, respectively. The 1-year OS and PFS were 69 and 59%, respectively. The same outcomes are reported separately in Table 2 for both MDR-colonized and not-colonized patients.

**Table 2:** Main outcomes whole population and colonized/not colonized cohorts.

	Whole population	Not colonized	Colonized
N	106	72	34
OS@1y	69%	69%	72%
PFS@1y	59%	60%	56%
NRM@100d	13%	15%	10%
NRM@1y	22%	25%	15%
G2-4 aGVHD	20%	21%	17%
G3-4 aGVHD	7%	7%	6%

OS, overall survival; PFS, progression-free survival; NRM, no relapse mortality; aGVHD, acute graft versus host disease.

### MDR-colonized cohort

**Figure 1:**
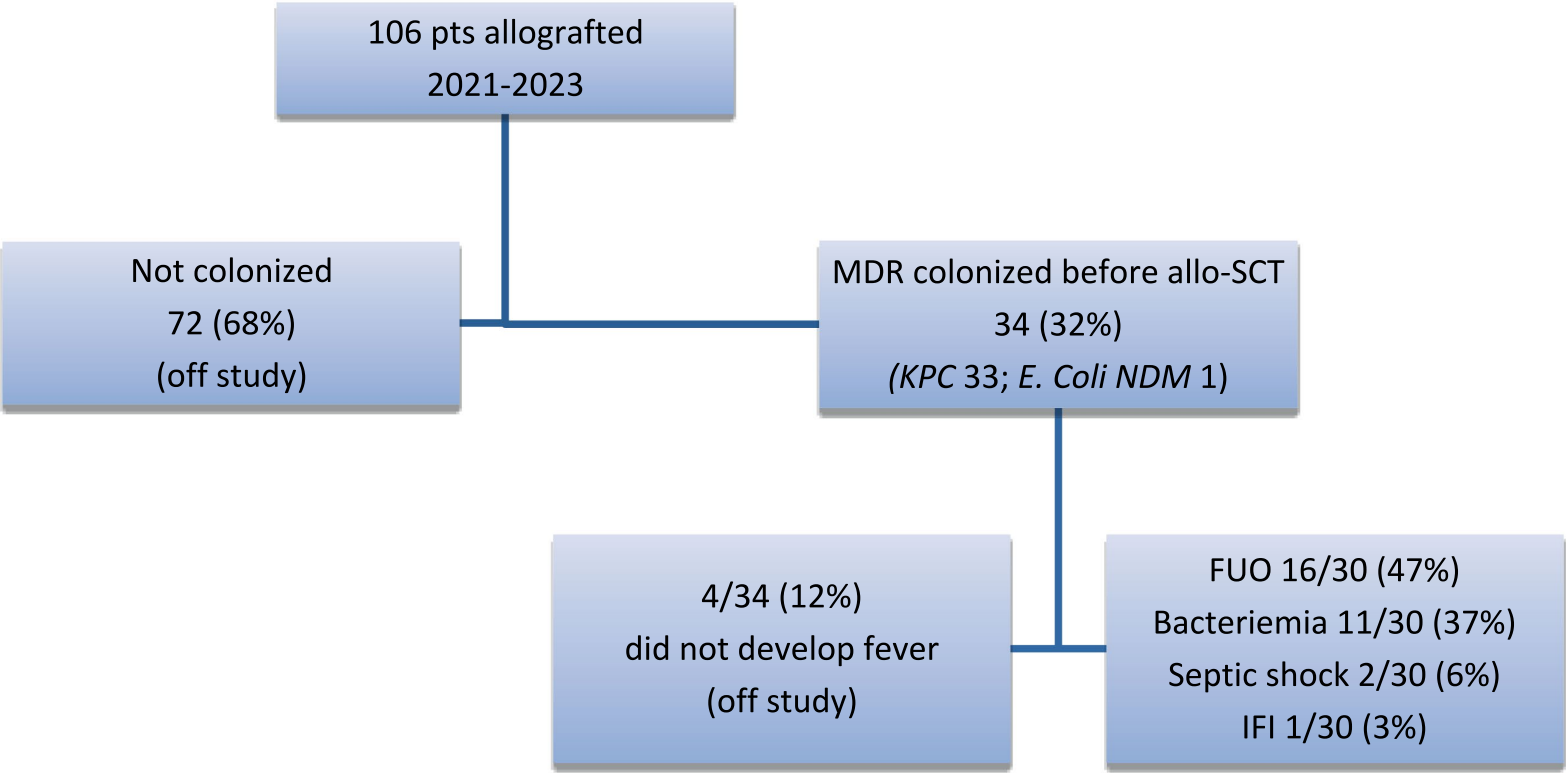
Flowchart of patients included in this study. MDR, multidrug-resistant; KPC, *klebsiella pneumoniae carbapemenase*; IFI, invasive fungal infection; NDM, New Delhi metallo-beta-lactamase 1.

Figure 1 shows the flowchart of the total number of patients included in this study. A total of 34 patients had MDR colonization at the time of allo-SCT or had a history of previous MDR-bacteria colonization/infection. Specifically, 18 (53%) of them were colonized based on rectal swabs taken at the time of hospitalization in the BMT Unit. All but one of these infections were caused by *KPC* (33), with one patient infected by *E. coli* carrying the NDM gene.

During the pre-engraftment period, 4 patients (12%) did not develop fever, while 16 (47%) developed fever of unknown origin (FUO). A total of 11 patients (32%) developed bacteremia: 7 (63%) were caused by gram-positive cocci, and 4 (37%) by gram-negative bacteria (*Escherichia Coli, Pseudomonas, Klebsiella pneumonia* sensible to carbapemenase, *and Klebsiella pneumonia ESBL*). Two patients went into a septic shock due to staph hominis and *Pseudomonas aeruginosa*. One patient developed an invasive fungal lung infection and was treated with voriconazole. Overall, no patients had *KPC* translocation. The EAT for patients during the first NFE is reported in Table 3. A total of 22 patients out of 30 (73%) received an EAT, which was active against MDR bacteria, while 8 patients out of 30 (27%) were treated with a conventional EAT. Among the latter, 7 patients out of 8 were not colonized at the time of hospitalization in the BMT unit. One of the patients, treated with meropenem at the time of the first NF, died at day +4 from septic shock induced by *Pseudomonas aeruginosa* sensitive to meropenem. In the whole cohort of patients, the de-escalation strategy was evaluated in 24 out of 30 (80%) patients (excluding 6 patients who received piperacillin–tazobactam as EAT). The rate of de-escalation was 79% (19 out of 24 patients).

**Table 3:** Empirical antibiotic therapy (EAT) in MDR patients.

EAT	*N* = 30
CAZAVI	5 (17%)
CAZAVI + aminoglycoside	10 (30%)
Cefiderocol	1 (3%)
MEVA	1 (3%)
MEVA + aminoglycoside	4 (13%)
MEVA + glycopeptide	1 (3%)
TAZO	5 (17%)
TAZO+ aminoglycoside	1 (3%)
Meropenem	2 (6%)

CAZAVI, ceftazidime + avibactam; MEVA, meropenem + vaborbactam; TAZO, piperacillin + tazobactam.

The all-cause mortality rates at 30 and 100 days for the whole MDR patient population were 6% (2/34) and 12% (4/34), respectively. At day 30, the cause of death was septic shock induced by *Pseudomonas aeruginosa* (day +4) and CMV pneumonia (day +46). On day 100, one patient relapsed and died, and one patient developed fatal neurological toxicity.

We analyzed the outcomes of patients with FUO to evaluate the feasibility and safety of the de-escalation strategy. Among the 16 patients with FUO, EAT was adapted to the resistance profile in 14 out of 16 patients (86%): 8 were treated with ceftazidime–avibactam, 5 with meropenem–vaborbactam, and 1 with cefiderocol. Two MDR-colonized patients were treated with piperacillin–tazobactam. Excluding these last two patients, the median duration of MDR-directed EAT was 3.5 days (range 2–8). The de-escalation rate was 75% (12 out of 16) and after de-escalation, no patients developed new episodes of bacteremia or sepsis by colonizing MDR bacteria. Only one patient died from disease relapse.

Finally, we analyzed the outcomes of patients with MDR-positive rectal swabs at the time of hospitalization in the BMT unit (18 patients, 56%). Their characteristics, reported in Table 1, had no significant differences when compared to the whole population. All patients but one developed fever during the aplasia period. Fever of unknown origin was present in 11 patients (65%), Five patients developed bacteremia: 3 due to gram-positive cocci, 1 from *Pseudomonas aeruginosa*, and 1 from ESBL *KP*. EAT was adapted to the resistance profile in 16 patients (94%), while one received piperacillin–tazobactam. Among 11 patients with FUO, 10 were treated with adapted EAT and one with piperacillin–tazobactam. Antibiotic de-escalation therapy was performed successfully in all patients, and the median duration of adapted EAT was 3 days (range 2–8). Day +30 and + 100 mortality rates were 0 and 10%, respectively. One patient died from disease relapse at day +100.

## Discussion

The outcomes of allo-SCT have progressively improved over the past few decades, mainly because of a decrease in the 1-year NRM from 29.7% in the 1980s to 12.2% in the 2010–2016 period. However, infections continue to account for a significant cause of death, with 20% of deaths attributed to infections in the more recent period, compared to 28% in the earlier years (Averbuch et al., 2013b). Severe infections due to bacteria, in particular the MDR gram-negative ones, represent an emerging problem in some countries. Indeed, as reported by the European CDC, in countries such as Italy the frequency of MDR *klebsiella pneumoniae* was almost 30%, remaining stable in the last few years.^1^

In hematological patients treated with intensive chemotherapy or transplantation inducing severe neutropenia and mucosal damage, the carriage of MDR bacteria in stool is considered a significant risk factor for translocation in the bloodstream, increasing the mortality rate. In a prospective study analyzing patients transplanted in Italy in 2014, it was shown that 3.4% of patients were colonized and had an increased risk of translocation during the pre-engraftment period, which resulted in a high risk of mortality (Girmenia et al., 2017). In ECIL-4 recommendations, a de-escalation approach was proposed in centers where MDR bacteria were frequently detected. In this situation, an active EAT with a known activity against MDR bacteria must be started, and when blood cultures return negative, narrower spectrum antibiotics can replace MDR-directed EAT (Averbuch et al., 2013a). This approach, however, was not tested in a high-risk population, such as the one undergoing allogeneic transplantation. Recently, Gustinetti et al. have described their de-escalation approach in 102 patients treated with allo-SCT. The rate of early and late de-escalation was 55.9% and was considered satisfactory. However, such patients were not colonized by MDR strains before allo-SCT (Gustinetti et al., 2018). Furthermore, a recent European survey revealed that in many transplant centers, the de-escalation approach is not considered a standard of care and is implemented only in 35% of the centers that responded to the survey (Verlinden et al., 2020).

In this retrospective study, we analyzed 106 allografted patients from 2021 to 2023. Of these patients, 34 (32%) were colonized or developed infections induced by MDR bacteria, and 18 (17%) were colonized in stool immediately before transplantation. Overall, the frequency of MDR colonization in our patients was considered dramatically higher than the one described in a prospective study from GITMO (Girmenia et al., 2017) and other retrospective studies where the pre-transplantation colonization rate was 4.8, 17, and 11%, respectively (De Rosa et al., 2017; Korula et al., 2020). Clearly, in colonized patients, the prompt initiation of EAT in case of fever during the pre-engraftment period is of utmost importance to reduce the risk of fatal outcome (Gutiérrez-Gutiérrez et al., 2017). According to the protocol at our center, all patients with proven colonization at the time of pre-transplantation work-up, or those with colonization or infection during the previous CT, were treated with an adapted MDR-driven EAT. However, in this cohort, only 84% received the treatment stated in the procedure. Some patients, particularly those not colonized by MDR strains during the pre-transplantation work-up, were instead treated with piperacillin–tazobactam.

The translocation rate during the pre-engraftment period of colonized patients was 32.5%, and the probability of 4-month survival was significantly lower in this cohort than in the not-colonized cohort (40% vs. 88%) (Girmenia et al., 2017). In this present study, KPC translocation was null. We can only speculate on the reasons for such a difference. The first possible explanation could be the intensity of conditioning regimens. Indeed, as per the transplant conditioning intensity score (TCI), which has been recently published (Spyridonidis et al., 2020), all the patients received intermediate and low TCI, which can reduce the risk of mucosal damage limiting the risk of translocation. Furthermore, for 10 patients, the Busulfan dose was adapted based on the AUC to avoid administering excessively toxic doses. The second possible explanation could be the use of GVHD prophylaxis, which included *in vivo* T-cell depletion (by ATG or post-transplantation cyclophosphamide) for all patients, regardless of donor type. This may have reduced the immunological attack on intestinal mucosa (Kröger et al., 2016; Bonifazi et al., 2019; Luznik et al., 2008). Finally, the well-known inverse relationship between resistance phenotype and fitness/virulence of bacteria could partially explain the low translocation rate observed (Vogwill and MacLean, 2015).

In the whole population of colonized patients, the rate of de-escalation to narrower ATB resulted feasible and safe. Indeed, in 79% of them, it was possible to de-escalate, and none died from MDR infection. The de-escalation strategy was also analyzed only in patients developing FUO. In this group, the rate of de-escalation to narrower ATB was 75%. As a consequence, the median duration of MDR-active antibiotic was 3.5 days. This figure is different when compared to other experiences. Micozzi et al. analyzed 55 patients colonized by *KPC* during the aplasia period after CT. Only 13% of them were allografted. The median duration of MDR-driven EAT was 12.8 days (range 7–29), while the median duration of fever was 2.8 days (range 1–9). The MDR-driven EAT was effective, and the attributable mortality rate was 2.1%. However, in this study, the de-escalation approach was not applied and was considered inappropriate due to the patient’s risk profile (Micozzi et al., 2023).

Although this retrospective study was carried out at a single retrospective center and has limitations due to its retrospective nature and small sample size, we can conclude that an antibacterial de-escalation strategy can be successfully applied in an MDR-colonized, deeply immunosuppressed population. This approach limits the duration of the therapy without affecting outcomes, notably the attributable mortality rate. It is also noteworthy that a low translocation rate was observed in these patients.

## Data Availability

The raw data supporting the conclusions of this article will be made available by the authors, without undue reservation.

## Footnotes

^1^ https://www.ecdc.europa.eu
